# Supplementary material for: Oestrogen Detoxification Ability of White Rot Fungus Trametes hirsuta LE-BIN 072: Exoproteome and Transformation Product Profiling
Source: J Fungi (Basel). 2024 Nov 15;10(11):795. doi: 10.3390/jof10110795 (PMC11595678; doi:10.3390/jof10110795)
Supplement: Supplementary file 1 [file jof-10-00795-s001.zip › jof-3293407-supplementary.pdf]

Supplementary Materials for Savinova O.S. et al. Oestrogen detoxification ability of white rot fungus *Trametes hirsuta* LE-BIN 072: exoproteome and transformation product profiling

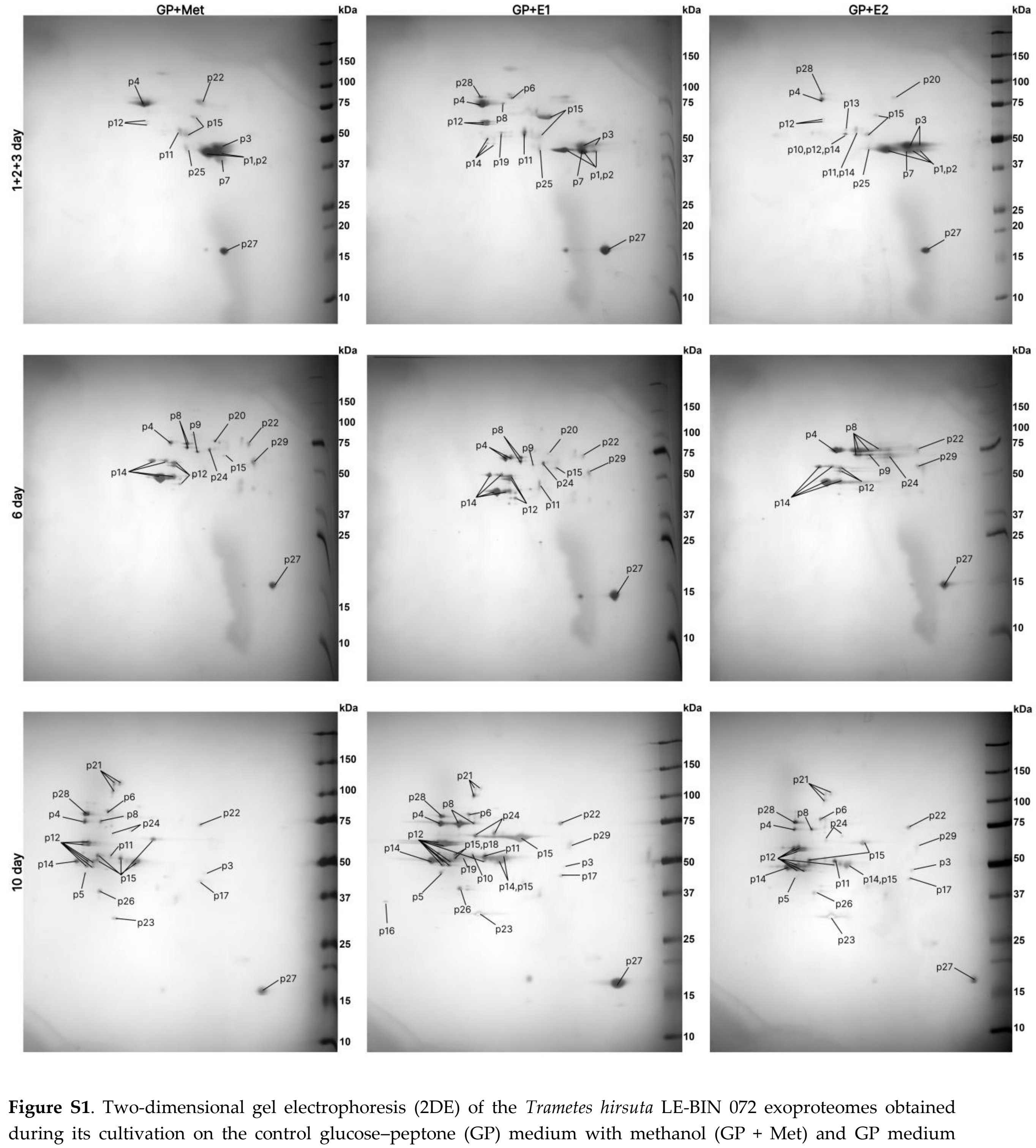

Figure S1. Two-dimensional gel electrophoresis (2DE) of the *Trametes hirsuta* LE-BIN 072 exoproteomes obtained during its cultivation on the control glucose–peptone (GP) medium with methanol (GP + Met) and GP medium supplemented with E1 (GP + E1) and E2 (GP + E2). Proteins secreted in GP + Met, GP + E1 and GP + E2 media are shown on 1 + 2 + 3 day of cultivation (top panel), the 6th day of cultivation (central panel) and the 10th day of cultivation (bottom panel). The designation "1 + 2 + 3 day" means that culture broths from days 1, 2 and 3 of cultivation were pooled together. For the data on the MALDI TOF/TOF MS/MS analyses of the highlighted protein spots, please refer to Figure 6.

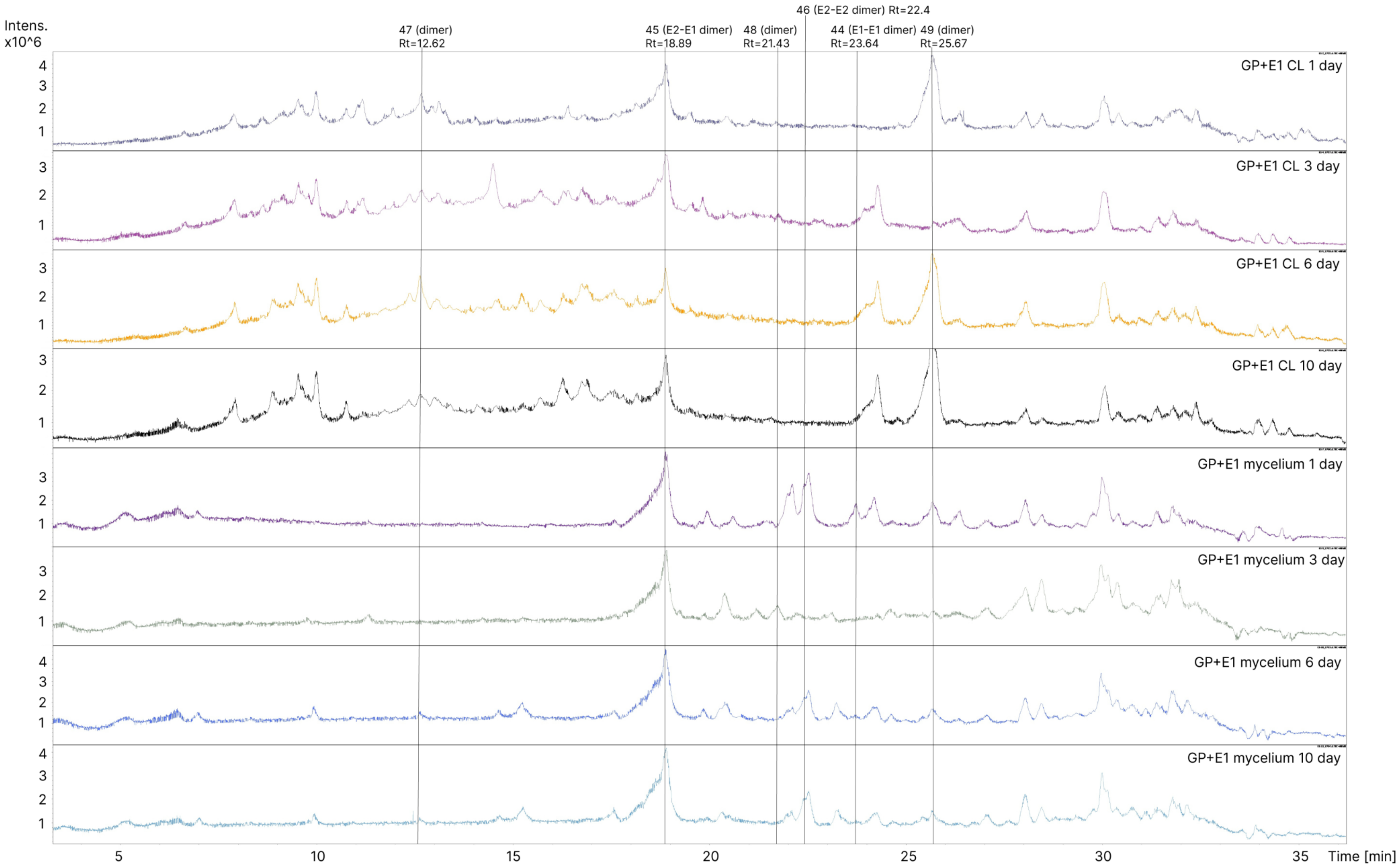

Figure S2. HPLC-MS chromatograms (TIC) of culture liquid (CL) and mycelium extracts of *Trametes hirsuta* LE-BIN 072 during cultivation on glucose–peptone (GP) medium supplemented with E1 (GP + E1). The designations of the compounds are presented in Table 1.

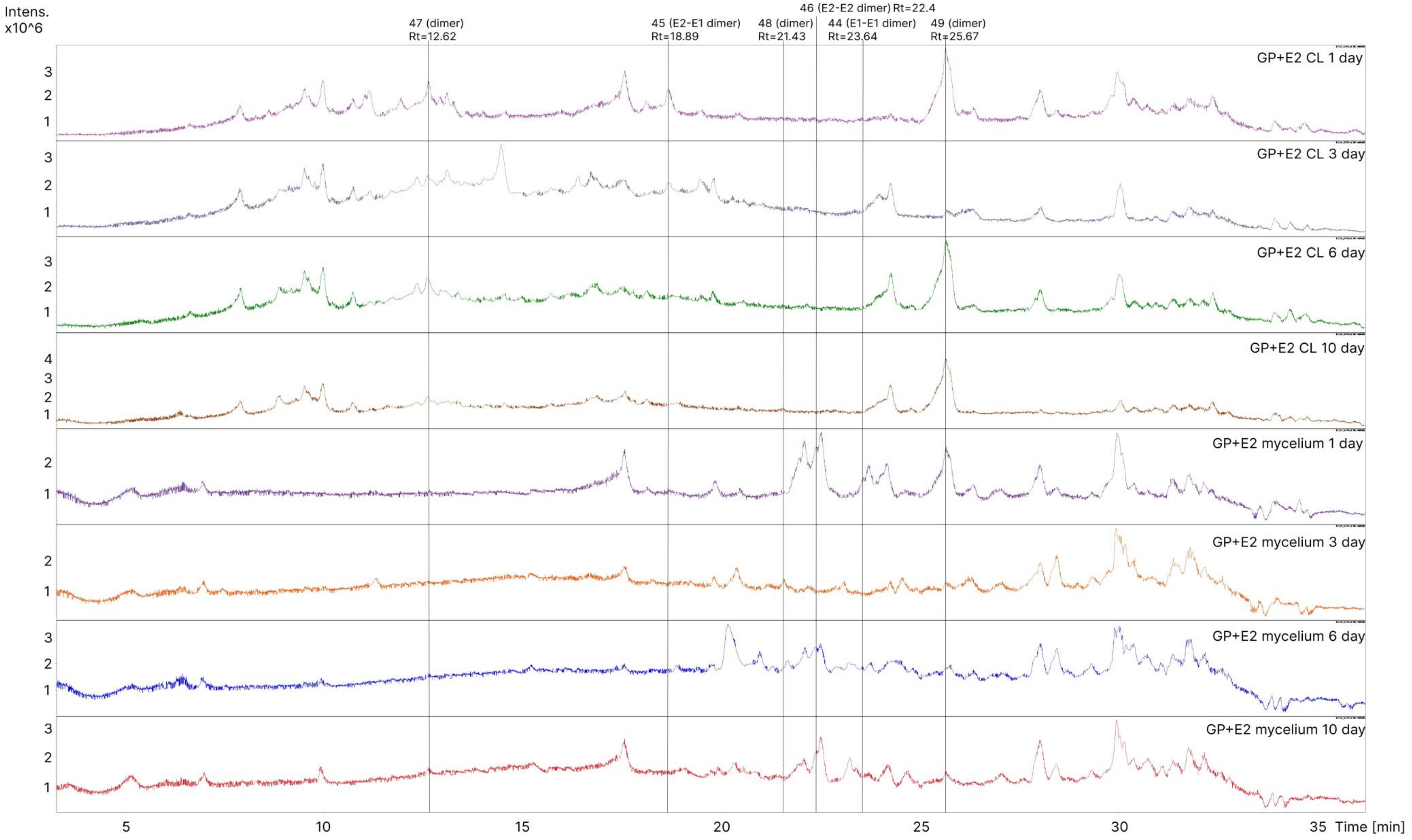

Figure S3. HPLC-MS chromatograms (TIC) of culture liquid (CL) and mycelium extracts of *Trametes hirsuta* LE-BIN 072 during cultivation on glucose–peptone (GP) medium supplemented with E2 (GP + E2). The designations of the compounds are presented in Table 1.
